# Depression, anxiety, and stress among urban and rural adolescents in Shivamogga, Karnataka

**DOI:** 10.12688/f1000research.139603.2

**Published:** 2024-05-21

**Authors:** Ajay Mallya, Raghavendraswamy Koppad, Praveen Kumar

**Affiliations:** 1 Department of Community Medicine, Kasturba Medical College, Mangalore, Manipal Academy of Higher Education, Manipal, India; 2 Department of Community Medicine, Shivamogga Institute of Medical Sciences, Shivamogga, Karnataka, 577201, India

**Keywords:** Mental health, Adolescence, Depression, anxiety, stress

## Abstract

**Background:** Currently there are 1.3 billion adolescents worldwide, which makes up 16% of the world population. Over 20% of adolescents around the world are thought to have behavioural or mental health issues. Addressing mental health issues is very important for the promotion of positive health in adolescents.

**Methods:** The objective of our study was to estimate the prevalence of depression, anxiety, and stress among adolescents in urban and rural areas of Shivamogga. A cross-sectional study was conducted among 350 adolescents aged 16 to 19 years each from urban and rural areas of Shivamogga.

**Results:** Depression, anxiety and stress were found to be 23.1%, 29.4% and 26.6% in urban areas and 19.1%, 24% and 21.1% in rural areas respectively.

**Conclusions:** About a quarter of the adolescent population suffers from depression anxiety and stress. Adopting and implementing better education and health policies are necessary to enhance adolescent mental health.

**Abbreviations**
DALYs: Disability Adjusted Life Years
DASS: Depression Anxiety Stress scale
SEAR: South East Asia Region
SPSS: Statistical Package for Social Sciences
WHO: World Health Organization

## Introduction

Currently, there are 1.3 billion adolescents worldwide, which makes up 16% of the world population. ^1^ Adolescents make up 20% of the population in countries in the South-East Asia region (SEAR) of the World Health Organization (WHO), and 21.8% of the Indian population. ^2^^,^^3^

Adolescence is a distinct period in human development and crucial for setting the groundwork for long-term health. Today nine out of ten adolescents are living in underdeveloped countries, where they confront particularly difficult obstacles in everyday lives from getting an education to simply surviving. ^4^ Over 20% of adolescents around the world are thought to have behavioural or mental health issues. Lifetime mental health disorders commonly begin by the age of 14, and depression is the leading cause of the global disease burden among adolescents. ^4^

Adolescents frequently suffer from emotional disturbances. Anxiety disorders predominate among adolescents and are more frequently seen in older adolescents compared to younger ones. Anxiety disorders are thought to affect 3.6% of 10–14-year-olds and 4.6% of 15–19-year-olds. ^5^ Depression and anxiety share some common features and together can result in poor performance in school and poor school attendance. ^5^ This, in addition to the marked social impairment experienced by depressed adolescents, will result in poor workplace relationships which in turn leads to poor connections and can affect one’s ability to make academic and workplace progress. Depression in adolescents also decreases the chance of them pursuing higher education and can also lead to lower employment rates. Depression in adolescents is also associated with earlier pregnancy and early parenthood. ^6^

Adolescents with depression run a very high risk of suicide. ^7^ Smoking, substance abuse, and obesity are all also more prevalent among adolescents who are depressed. ^8^^,^^9^ Depressive disorders are among the top three causes of adolescent disability adjusted life years (DALYs) lost globally and anxiety happens to be among the top five causes of loss of DALYs among adolescent girls. ^10^ About 7% of adolescents between the ages of 13 and 17 suffer from diagnosed mental health disorders in India according to the National Mental Health Survey 2015-16. ^11^

Stress in adolescents also affects their academic performance. ^12^ Levels of stress experienced are shown to increase from preadolescence to adolescence. ^13^ Studies have shown that girls tend to experience higher levels of stress compared to boys during adolescence. ^14^ There is evidence that shows a positive correlation between stress and depression and anxiety. ^15^

Addressing mental health issues is very important for the promotion of positive health in adolescents. It also facilitates a whole generation of adults with sound mental health and leads to a healthy society. And as we couldn’t identify any studies done on mental health issues in this region of Karnataka, there is a need to carry out research and gather more data and facilitate the promotion of positive mental health among adolescents in this part of Karnataka.

## Methods

### Ethics and consent

Ethical approval was granted by the Institutional Ethics Committee of Shivamogga Institute of medical Sciences, Shivamogga (ref no. SIMS/IEC/414) on 09/11/2018. Informed consent was taken from study participants and the parents/guardians in case of minors (16-17 years) via Google Forms. Assent was also taken from study participants of age 16-17 years.

### Study design and data collection

A cross-sectional study was conducted in urban and rural areas of Shivamogga taluk to estimate the prevalence of depression, anxiety, and stress among adolescents. This study was conducted from August 2020 to September 2020. The study population is composed of all adolescent males and females aged 16-19 years in rural and urban field areas of Shivamogga taluk. According to an Indian study, the prevalence (p) of anxiety among adolescents was found to be 24.4%. ^16^ With desired absolute precision of 5% the minimum sample size required was calculated using the formula Z∞ ^2^pq/D ^2^. With this, we got a sample size of 295 adolescents. Considering 10% as non-respondents, the final sample size was 325 which was rounded off to 350. So, 350 samples were collected in urban and rural areas each.

The Block education officer was approached and a list of all pre-university colleges in Shivamogga taluk was collected. Shivamogga taluk has 36 pre-university colleges in the urban area and 10 pre-university colleges in the rural area. Then 50% of the pre-university colleges were randomly selected using the random number table both in urban and rural areas. So, 18 colleges from the urban and 5 colleges from the rural area were selected. From each selected colleges, a class was randomly selected. For each selected class the lottery method was used to select 20 students from each class in the urban area and 70 students from the rural area until the desired sample size was reached. All adolescents in the age group of 16 to 19 years were included in the study. Adolescents who did not fill out the questionnaire completely were excluded from the study.

After taking consent, a questionnaire that included basic demographic details (age and sex) with the DASS 21 scale (Depression Anxiety Stress Scale) ^17^ in the English without any translation or alteration was used to collect data. Given that English is the primary language of instruction at the PU college, we employed the scale without any need for translation. A Google Form of the questionnaire was created, one each for urban and rural areas and the link to fill out the form was shared with students willing to participate in the study via ‘WhatsApp’ mobile application. Adolescents aged 16 completed years and above until the age of 19 were included in the study.

### Statistical analysis

Data was entered into a Microsoft Excel spreadsheet. SPSS (Version 21) was used to perform statistical analysis. Data analysis was done using appropriate statistical tools. Results were expressed in terms of frequency, percentages, Chi-square values, and P-values. All statistical tests were performed with a confidence level of 95% and a power of 80%. In the significance tests, P value equal to or less than 0.05 was considered as statistically significant.

## Results

As shown in Table 1: a total of 700 adolescents participated in the study. Out of which 350 (50%) adolescents were from urban areas and another 350 (50%) adolescents were from rural areas. Males and females in urban areas were 153 (44%) and 197 (56%) respectively. In rural areas, males and females were 191 (55%) and 159 (45%) respectively.

**Table 1.** Distribution of study participants according to depression, anxiety and stressed in urban and rural areas. (n=700).

Sl. No.	Characteristics	Urban	Rural	P value
1	No. of participants			0.004
	• Males	153 (44%)	191 (55%)	
	• Females	197 (56%)	159 (45%)	
2	Age of the participants			0.011
	• 16 years	94 (27%)	60 (17%)	
	• 17 years	142 (40.6%)	159 (45%)	
	• 18 years	100 (29%)	121 (35%)	
	• 19 years	14 (4%)	19 (3%)	
3	Depression			0.195
	• Mild	25 (7.1%)	32 (9.1%)	
	• Moderate	46 (13.1%)	28 (8%)	
	• Severe	10 (2.9%)	7 (2%)	
	• Not depressed	269 (76.9%)	283 (80.9%)	
4	Anxiety			0.105
	• Mild	29 (8.2%)	21 (6%)	
	• Moderate	71 (20.3%)	58 (16.6%)	
	• Severe	3 (0.9%)	5 (1.4%)	
	• Not anxious	247 (70.6%)	266 (76%)	
5	Stress			0.092
	• Mild	63 (18%)	54 (15.4%)	
	• Moderate	24 (6.9%)	13 (3.7%)	
	• Severe	6 (1.7%)	7 (2%)	
	• Not stressed	257 (73.4%)	276 (78.9%)	

All our study participants were aged between 16 to 19 years. the mean age of the participants in the urban area was 17.10±0.84 years. In the urban area, the majority of the participants (142) were aged 17 years (40.6%). Participants aged 16 years were 94 (27%), 18 years were 100 (29%) and 19 years were 14 (4%). Similarly in rural areas, the mean age of the participants was 17.10 ±0.76 years. The majority (159) of the participants were aged 17 years (45%). Participants aged 16 years were 60 (17%), 18 years were 121 (35%) and 19 years were 10 (3%).

Among the adolescents in urban areas, 23.1% were depressed, 29.4% had anxiety and 26.6% had stress. Similarly, among the adolescents in rural areas, 19.1% were depressed, 24% had anxiety and 21.1% were stressed.

Among the depressed adolescents, in urban areas the majority (46; 13.1%) had moderate depression, whereas in rural areas the majority (32; 9.1%) had mild depression. Among the adolescents who had anxiety, both in urban and rural areas the majority (71 (20.3%) and 58 (16.6%) respectively) had moderate anxiety. Among the adolescents who were stressed, both in urban and rural areas, the majority (63 (18%) and 54 (15.4%) respectively) had mild stress.

Among the study participants from urban areas of Shivamogga, 33 (21.6%) adolescent males and 48 (24.4%) adolescent females were depressed, 33 (21.6%) adolescent males and 70 (35.5%) adolescent females were anxious, and 55 (35.9%) adolescent males and 38 (19.3) adolescent females were stressed (see Table 2). A significantly greater number of females were anxious and stressed than males.

**Table 2.** Distribution of study participants based on sex in urban areas of Shivamogga (n=350).

Sl. No.	Characteristics	Males	Females	P value
1	Not depressed	120 (78.4%)	149 (75.6%)	0.538
	Depressed	33 (21.6%)	48 (24.4%)	
2	Not anxious	120 (78.4%)	127 (64.5%)	0.005
	Anxious	33 (21.6%)	70 (35.5%)	
3	Not stressed	98 (64.1%)	159 (80.7%)	<0.001
	Stressed	55 (35.9%)	38 (19.3%)	

Among the study participants from rural areas of Shivamogga, 26 (13.6%) adolescent males and 41 (25.8%) adolescent females were depressed, 40 (20.9%) adolescent males and 44 (27.7%) adolescent females were anxious, and 47 (24.6%) adolescent males and 27 (17%) adolescent females adolescents were stressed. A significantly greater number of females were stressed than males; see Table 3.

**Table 3.** Distribution of study participants based on sex in rural areas of Shivamogga (n=350).

Sl. No.	Characteristics	Males	Females	P Value
1	Not depressed	165 (86.4%)	118 (74.2%)	0.004
	Depressed	26 (13.6%)	41 (25.8%)	
2	Not anxious	151 (79.1%)	115 (72.3%)	0.167
	Anxious	40 (20.9%)	44 (27.7%)	
3	Not stressed	144 (75.4%)	132 (83%)	0.089
	Stressed	47 (24.6%)	27 (17%)	

## Discussion

Our study attempted to find out the prevalence of depression, anxiety, and stress among adolescents in urban and rural areas. The current study found that the prevalence of depression was 23.13% and 19.14% in urban and rural areas respectively. On average, depression among adolescents in Shivamogga is 21.14%. Similar results were found by Kumar K S et al. and Sahoo S, where depression among adolescents was 19.5%. and 18.5% respectively. ^16^^,^^18^ Depression was seen to be higher in females in both urban and rural areas. The difference was statistically significant in rural areas of Shivamogga. Similar observations were made in other studies across India where depression was seen to be higher among female respondents. ^16^^,^^19^^,^^20^ Genetic predisposition and hormonal influences could be the reason why depression was higher in females than males. This could also be due to higher reporting of symptoms by females, whereas fewer males may report symptoms due to societal pressure for males to be more emotionally strong.

Depression was more frequently seen in urban adolescents than rural adolescents in the current study (23.1% and 19.4% respectively). Similar observations were made by Bahl R, and Kumari R in their study, where depression among urban adolescents was about 33.3% and depression among rural adolescents was 20.7%. ^21^ This could be due to higher exposure of adolescents to risk factors like academic competition and failure, anxiety, and substance abuse, etc.

Anxiety among adolescents in the current study was found to be 29.2% and 24% in urban and rural areas of Shivamogga respectively. On average, anxiety was found to be 26.6%. This was similar to the study conducted by Kumar K S et al. and Madasu S et al., who found anxiety among adolescents to be 24.4% and 22.7% respectively. ^16^^,^^22^ These studies also found that the prevalence of anxiety was higher among females when compared to males. Similar findings were seen in our study in both urban and rural areas. The prevalence of anxiety was significantly higher among females than males in urban areas. Higher anxiety among females could be due to higher exposure to risk factors like academic competition and societal pressure on women to work and prove themselves as equals to males in the modern world.

Anxiety was more frequently seen in urban adolescents than rural adolescents in our study (29.4% and 24% respectively). Similar results were observed by Vs P et al. ^23^ This could be due to more academic competitiveness in an urban area than in a rural area. While the parental expectation and societal pressure to perform better academically and excel in the future and have a well-paying job is higher for males than females, in the modern world the same pressure is higher in females as well in urban areas. This could be the reason why anxiety was more prevalent among urban females than males. As this study was conducted during the pandemic, the increased awareness of COVID in urban areas with better education could also be the reason why anxiety was higher among urban adolescents.

Stress among the adolescents in our study was found to be 26.6% and 21.1% in urban and rural areas respectively. On average 23.85% of the adolescents were stressed in Shivamogga. Sahoo and Saddicha, in their study, also found that the prevalence of stress among adolescents was 20%. ^18^ Prevalence of stress was found to be higher in urban areas compared to rural areas. Similar observations were made by Dey BK ^24^ in Bangladesh. No studies from the Indian setting could be found that compared the prevalence of stress in urban and rural areas.

Stress was more frequently seen in males than females in both urban and rural areas of Shivamogga. This was contrary to observations made in other studies, where they found that stress was more prevalent among female participants. ^16^^,^^24^ This difference could be due to underreporting of symptoms by female participants. The ongoing pandemic at the time of the study could have contributed to these findings.

### Strengths and limitations

The strength of the study lies in its robust methodology, extensive sample size and comprehensive data analysis. This study employed a well-designed survey or assessment tool to gather data, ensuring the accuracy and reliability of the results. The measurement of depression, anxiety, and stress was conducted using a standardized scale rather than through assessment by a trained psychiatrist.

While we ensured that the collected data is properly segregated into urban and rural categories based on the list of colleges obtained from the block education officer, it is worth noting that some participants from rural areas may commute and study in a college situated in urban areas, and vice versa.

## Conclusions

About a quarter of the adolescent population suffers from depression anxiety and stress. It’s crucial to detect these symptoms among adolescents and take proper action to avoid compromising their education and development. Those detected to have these symptoms must be referred to be seen by a psychiatrist for clinical evaluation. Further studies are necessary to learn about the causative factors of these mental health disorders. Adopting and implementing better education and health policies are necessary to enhance adolescent mental health.

## Data Availability

figshare: DASS.xlsx. https://doi.org/10.6084/m9.figshare.23652477.v2 ^25^ This project contains the underlying data file. figshare: Questionnaire - Depression, anxiety, and stress among adolescents in Shivamogga, Karnataka. https://doi.org/10.6084/m9.figshare.23996670.v2. ^26^ This project contains the blank questionnaire. Data are available under the terms of the Creative Commons Attribution 4.0 International license (CC-BY 4.0).

## References

[1] Adolescents Statistics: UNICEF DATA. [cited 2023 Apr 1]. Reference Source

[2] Adolescent health:[cited 2023 Apr 1]. Reference Source

[3] Behal S Debarati M Oommen K : Investing in Adolescent Health: Harnessing India’s Demographic Dividend. *ORF.* [cited 2023 Apr 1]. Reference Source

[4] UNICEF , editor. *Adolescence: an age of opportunity.* New York, NY: UNICEF;2011;138p. (The state of the world’s children).

[5] Mental health of adolescents:[cited 2023 Apr 1]. Reference Source

[6] Systematic Review and Meta-Analysis: Adolescent Depression and Long-Term Psychosocial Outcomes - ClinicalKey. [cited 2023 Apr 1]. Reference Source 10.1016/j.jaac.2018.07.896 30577941

[7] Windfuhr K While D Hunt I : Suicide in juveniles and adolescents in the United Kingdom. *J. Child Psychol. Psychiatry.* 2008;49(11):1155–1165. 10.1111/j.1469-7610.2008.01938.x 19017029

[8] Hasler G Pine DS Kleinbaum DG : Depressive symptoms during childhood and adult obesity: the Zurich Cohort Study. *Mol. Psychiatry.* 2005 Sep 1;10(9):842–850. 10.1038/sj.mp.4001671 15838533

[9] Keenan-Miller D Hammen CL Brennan PA : Health Outcomes Related to Early Adolescent Depression. *J. Adolesc. Health.* 2007 Sep;41(3):256–262. 10.1016/j.jadohealth.2007.03.015 17707295 PMC2034364

[10] World Health Organization: *Global accelerated action for the health of adolescents (AA-HA!): guidance to support country implementation.* Geneva: World Health Organization;2017 [cited 2023 Apr 1];176. Reference Source

[11] NIMHANS: *National Mental Health Survey of India, 2015-16: Mental Health Systems.* National Institute of Mental Health and Neuro Sciences;145. Reference Source

[12] Moksnes UK Moljord IEO Espnes GA : The association between stress and emotional states in adolescents: The role of gender and self-esteem. *Personal. Individ. Differ.* 2010 Oct 1;49(5):430–435. 10.1016/j.paid.2010.04.012

[13] Rudolph KD : Gender differences in emotional responses to interpersonal stress during adolescence. *J. Adolesc. Health.* 2002 Apr;30(4):3–13. 10.1016/S1054-139X(01)00383-4 11943569

[14] Arsenio WF Loria S : Coping with Negative Emotions: Connections with Adolescents’ Academic Performance and Stress. *J. Genet. Psychol.* 2014 Jan 1;175(1):76–90. 10.1080/00221325.2013.806293 24796156

[15] Sex Differences in Adolescent Depression: Stress Exposure and Reactivity Models - Hankin - 2007 - Child Development - Wiley Online Library. [cited 2023 Apr 17]. 10.1111/j.1467-8624.2007.00997.x 17328705

[16] Kumar KS Akoijam BS : Depression, Anxiety and Stress Among Higher Secondary School Students of Imphal, Manipur. *Indian J. Community Med.* 2017;42(2):94–96. 10.4103/ijcm.IJCM_266_15 28553025 PMC5427869

[17] Depression Anxiety Stress Scales (DASS):[cited 2023 Sep 8]. Reference Source

[18] Sahoo S Khess CRJ : Prevalence of Depression, Anxiety, and Stress Among Young Male Adults in India: A Dimensional and Categorical Diagnoses-Based Study. *J. Nerv. Ment. Dis.* 2010 Dec;198(12):901–904. 10.1097/NMD.0b013e3181fe75dc 21135643

[19] Singhal M Manjula M Vijay Sagar KJ : Subclinical depression in Urban Indian adolescents: Prevalence, felt needs, and correlates. *Indian J. Psychiatry.* 2016;58(4):394–402. 10.4103/0019-5545.196727 28196996 PMC5270264

[20] Mishra SK Srivastava M Tiwary NK : Prevalence of depression and anxiety among children in rural and suburban areas of Eastern Uttar Pradesh: A cross-sectional study. *J. Family Med. Prim. Care.* 2018;7(1):21–26. 10.4103/jfmpc.jfmpc_248_17 29915728 PMC5958570

[21] Bahl R Kumari R : Mental Health Profile of Urban and Rural Adolescents in Jammu District of J&K. 2016;18:3.

[22] Madasu S Malhotra S Kant S : Anxiety Disorders among Adolescents in a Rural Area of Northern India using Screen for Child Anxiety-Related Emotional Disorders Tool: A Community-based Study. *Indian J. Community Med.* 2019;44(4):317–321. 10.4103/ijcm.IJCM_359_18 31802792 PMC6881902

[23] Prabha VS Rao VB Kanakabushanam GVVS : A comparative study of anxiety and depression among adolescents from rural and urban areas. *J. Med. Sci. Res.* 2017 Jan 3;5(1):29–32. 10.17727/JMSR.2017/5-6

[24] Dey BK Rahman A Bairagi A : Stress and Anger of Rural and Urban Adolescents. *Psychology.* 2014 Mar 18 [cited 2023 Apr 23];2014. Reference Source

[25] Mallya A : Responses - Depression, anxiety, and stress among adolescents in Shivamogga, Karnataka.[Dataset]. *figshare.* 2023. 10.6084/m9.figshare.23652477.v2 PMC11211729 38948506

[26] Mallya A : Questionnaire - Depression, anxiety, and stress among adolescents in Shivamogga, Karnataka.Dataset. *figshare.* 2023. 10.6084/m9.figshare.23996670.v2 PMC11211729 38948506

