# Supplementary material for: Depression, anxiety, and stress among urban and rural adolescents in Shivamogga, Karnataka
Source: F1000Res. 2024 Jul 12;12:1583. Originally published 2023 Dec 14. [Version 3] doi: 10.12688/f1000research.139603.3 (PMC11211729; doi:10.12688/f1000research.139603.3)
Supplement: Peer review history

# Reviewer report: Reviewer response for version 3

**DOI:** 10.5256/f1000research.168246.r302320

**Published:** 2024-07-13

**Authors:** Mohammad Meshbahur Rahman

**Affiliations:** 1 Department of Biostatistics, National Institute of Preventive and Social Medicine, Dhaka, Bangladesh

The authors has improved the manuscript. But there are some additional comments that can improve the quality of the manuscript. 

 1. In abstract, the methods section is still needs to improve. The sample size is 700 (350 from both urban and rural). Therefore, revise the first sentence of the methods. In addition, please report the study time, questionnaire tool (DAS) in one sentence. Finally, report the data analysis and software use information in third and last sentence. The author can follow the Methods section of abstract of the paper of Das et al., 2023 [Ref-1].

 2. The author reported that "Depression was more common among females in both urban and rural settings, with a significant difference observed in rural areas. Anxiety and stress were also more prevalent among females, with anxiety significantly higher in urban females. Urban adolescents exhibited higher levels of depression, anxiety, and stress compared to their rural counterparts." This findings has wrongly interpreted. We can not compare the findings without performing inferential statistics like t-test, proportion z-test or regression. Therefore, this article needs expert statistical assistance. The authors can consult to a statistician to improve this part.

 In the full-text article, the sample size estimation formula is not correctly reported. The authors can follow the Methods section of the work of Das et al. (2023) [ Ref-1] mentioned above.

 3. The discussion of the manuscript still needs substantial improving.

 I hope the above comments will help to improve the manuscript further.

Is the work clearly and accurately presented and does it cite the current literature?

Partly

If applicable, is the statistical analysis and its interpretation appropriate?

Partly

Are all the source data underlying the results available to ensure full reproducibility?

Partly

Is the study design appropriate and is the work technically sound?

Partly

Are the conclusions drawn adequately supported by the results?

Partly

Are sufficient details of methods and analysis provided to allow replication by others?

No

Reviewer Expertise:

Public Health, Global Health, Biostatistics, Epidemiology

I confirm that I have read this submission and believe that I have an appropriate level of expertise to confirm that it is of an acceptable scientific standard, however I have significant reservations, as outlined above.

## References

[1] : Exploring the knowledge and practices on road safety measures among motorbikers in Dhaka, Bangladesh: a cross-sectional study. *Inj Prev* .2023; 10.1136/ip-2023-045071 10.1136/ip-2023-045071 38050086 PMC12322459

# Reviewer report: Reviewer response for version 2

**DOI:** 10.5256/f1000research.164841.r287888

**Published:** 2024-06-27

**Authors:** Saranya Rajamanickam

**Affiliations:** 1 PSG Institute of Medical Sciences & Research, Coimbatore, Tamil Nadu, India

Methodology:

 1. Operational definition of dependent variable such as depression, stress and anxiety can be mentioned.

 2. Type of sampling can be mentioned.

 3. Need for separate google forms for urban and rural area can be explained.

 4. Scoring of depression, anxiety and stress to categorize it into mild, moderate an severe is not clear.

 Result:

 5. A table depicting the frequency of depression, anxiety and stress among the urban and rural area explaining the objective will be appreciated.

 6.  Significant p value can be highlighted in all the tables and   the level of  confidence interval can be written below each table.

**Competing Interests:** Nil Close

Is the work clearly and accurately presented and does it cite the current literature?

Yes

If applicable, is the statistical analysis and its interpretation appropriate?

Yes

Are all the source data underlying the results available to ensure full reproducibility?

Yes

Is the study design appropriate and is the work technically sound?

Yes

Are the conclusions drawn adequately supported by the results?

Yes

Are sufficient details of methods and analysis provided to allow replication by others?

Partly

Reviewer Expertise:

NCDs,

I confirm that I have read this submission and believe that I have an appropriate level of expertise to confirm that it is of an acceptable scientific standard, however I have significant reservations, as outlined above.

# Reviewer report: Reviewer response for version 2

**DOI:** 10.5256/f1000research.164841.r287883

**Published:** 2024-06-24

**Authors:** Rohith Motappa

**Affiliations:** 1 Department of Community Medicine, Kasturba Medical College, Kasturba Medical College Hospital, Mangalore, Manipal Academy of Higher Education, Karnataka, Manipal, Karnataka, Manipal, 576 104, India

This cross-sectional study aimed to assess the extent of depression, anxiety, and stress among adolescents residing in urban and rural areas of Shivamogga Taluk. Data was gathered over August and September 2020 through surveys involving 700 teens aged 16 to 19.

 The results showed that these mental health issues were common in both urban and rural adolescents, with higher rates observed in urban areas. Females consistently showed rates across all mental health conditions.

 The author should consider expanding on the following areas:

 1. If a selected student was absent during the data collection, how was the situation handled to maintain the desired sample size? What specific steps were taken in such cases?

 2. The author may include the implications of this study in the discussion.

 3. The author may also include some additional limitations of the study.

Is the work clearly and accurately presented and does it cite the current literature?

Yes

If applicable, is the statistical analysis and its interpretation appropriate?

Yes

Are all the source data underlying the results available to ensure full reproducibility?

Yes

Is the study design appropriate and is the work technically sound?

Yes

Are the conclusions drawn adequately supported by the results?

Partly

Are sufficient details of methods and analysis provided to allow replication by others?

Partly

Reviewer Expertise:

Non-Communicable Diseases, Mental Health, Geriatrics, Tuberculosis.

I confirm that I have read this submission and believe that I have an appropriate level of expertise to confirm that it is of an acceptable scientific standard.

# Reviewer report: Reviewer response for version 2

**DOI:** 10.5256/f1000research.164841.r287885

**Published:** 2024-06-11

**Authors:** Mohammad Meshbahur Rahman

**Affiliations:** 1 Department of Biostatistics, National Institute of Preventive and Social Medicine, Dhaka, Bangladesh

I want to thank the editor for inviting me to review the study. The sample size of this study is sufficient and the study design is appropriate. However, the study methods is not technically sound. The quire from the reviewer are pointed out below:

 1. This was a very simple study where the authors just assessed DAS among 350 adolescents. There is a major limitation of demographic variables in this study. There are many factors that affects DAS of adolescents. 

 2. The study just presented frequency distribution, and no inferential statistics has presented. 

 3. The abstract of the study is insufficient and lack of key information. In abstract, the authors reported their study objectives in methods section. But, it should be place at the end of Background/Introduction section. In the methods section, the authors should report about DAS 21 and its' computational ways, and appropriate statistical analysis. 

 4. The results section in abstract is incomplete. The author should report associated factors of DAS among adolescents. 

 5. In the main text, the writings of introduction is very poor. The author are requested to add the recent literature and statistics in the introduction, avoiding orphan paragraphs. The introduction would be sound if the author would report the DAS of adolescents in the similar socio-demographics in South-Asia. [Ref.: Siddik M, et al., 2024 [Ref- 1];  Mridha MK et al., 2021 [Ref- 2]; Singh MM, et al., 2017 [Ref-3]; Bhattarai D, et al., 2020 [Ref-4]]

 6. The methods of the study is very poor. The sample size estimation formula should write appropriately. The should report the scaling and scoring techniques of DAS 21. 

 7. To enrich the Methods of the study, the authors are requested to follow any standard approach of writing Methods [e.g,. Haque M, et al., 2023 [Ref- 5]; Siddik M, et al., 2024 [Ref-1]]. 

 8. In the results section, the authors are strongly recommended to add inferential statistics to identify the associated risk factors of DAS among adolescents.

 9. The discussion should be revised according to the new results.

Is the work clearly and accurately presented and does it cite the current literature?

Partly

If applicable, is the statistical analysis and its interpretation appropriate?

Partly

Are all the source data underlying the results available to ensure full reproducibility?

Partly

Is the study design appropriate and is the work technically sound?

Partly

Are the conclusions drawn adequately supported by the results?

Partly

Are sufficient details of methods and analysis provided to allow replication by others?

No

Reviewer Expertise:

Public Health, Global Health, Biostatistics, Epidemiology

I confirm that I have read this submission and believe that I have an appropriate level of expertise to state that I do not consider it to be of an acceptable scientific standard, for reasons outlined above.

## References

[1] : Physio-psychosocial risk of depression among college-going adolescents: A cross-sectional study in Bangladesh. *Journal of Affective Disorders Reports* .2024;16: 10.1016/j.jadr.2024.100768 10.1016/j.jadr.2024.100768

[2] : Prevalence and associated factors of depression among adolescent boys and girls in Bangladesh: findings from a nationwide survey. *BMJ Open* .2021;11(1) : 10.1136/bmjopen-2020-038954 e038954 10.1136/bmjopen-2020-038954 33455924 PMC7813352

[3] : Prevalence & factors associated with depression among schoolgoing adolescents in Chandigarh, north India. *Indian J Med Res* .2017;146(2) : 10.4103/ijmr.IJMR_1339_15 205-215 10.4103/ijmr.IJMR_1339_15 29265021 PMC5761030

[4] : Prevalence and factors associated with depression among higher secondary school adolescents of Pokhara Metropolitan, Nepal: a cross-sectional study. *BMJ Open* .2020;10(12) : 10.1136/bmjopen-2020-044042 e044042 10.1136/bmjopen-2020-044042 33384401 PMC7780534

[5] : Prevalence of and factors associated with depression, anxiety and stress among adolescents in Bangladesh during the COVID-19 pandemic: A population-based study. *F1000Research* .2023;12: 10.12688/f1000research.132304.1 10.12688/f1000research.132304.1 37476505

# Response

**Published:** 2024-06-21

**Authors:** Ajay Mallya

**Affiliations:** Community Medicine, Kasturba Medical College, Mangalore, karnataka, India

**F1000 research article response**

  

- **Comment: **This was a very simple study where the authors just assessed DAS among 350 adolescents. There is a major limitation of demographic variables in this study. There are many factors that affects DAS of adolescents.

**Author’s Response: **The study was conducted on 700 adolescents with 350 adolescents in urban and rural areas respectively. The sample size estimation for this study is clearly mentioned in the methodology and the number was not chosen arbitrarily. We have already agreed that measurement of depression, anxiety and stress among adolescents with a standard scale and not by a trained psychiatrist is a limitation of the study. Study of factors that affect DAS among adolescents was not in the scope of this study. This may be incorporated into future studies.

  

- **Comment: **The study just presented frequency distribution, and no inferential statistics has presented.

**Author’s Response: **We believe that presenting the frequency distribution serves as a critical first step in understanding the scope of these mental health issues in the region. It offers a clear and immediate representation of the data, which is particularly valuable for an area that has been under-researched in this context. While we acknowledge the value of inferential statistics in drawing broader conclusions from the data, the primary aim of our study was to highlight the prevalence rates and lay the groundwork for future research that may include more sophisticated statistical analyses. In future studies, we plan to incorporate inferential statistics to explore the relationships between various demographic variables and DAS among adolescents.

  

- **Comment:** The abstract of the study is insufficient and lack of key information. In abstract, the authors reported their study objectives in methods section. But it should be place at the end of Background/Introduction section. In the methods section, the authors should report about DAS 21 and its' computational ways, and appropriate statistical analysis.

**Author’s Response: **We agree with your comment. The suggested correction has been made.

**Action taken in the manuscript: **We have added more information in the result section of the abstract. The aim of the study is now placed at the end of the Introduction section. The same has been incorporated into the introduction and methodology sections of the main article as well. We have also added details of DAS calculation in the methodology section.

  

- **Comment:** The results section in abstract is incomplete. The author should report associated factors of DAS among adolescents.

**Author’s Response:  **Study of factors affecting DAS was not in the scope of our study. We will try to incorporate this into future studies in the area.

  

- **Comment:** In the main text, the writings of introduction is very poor. The author are requested to add the recent literature and statistics in the introduction, avoiding orphan paragraphs. The introduction would be sound if the author would report the DAS of adolescents in the similar socio-demographics in South-Asia. [Ref.: Siddik M, et al., 2024 [Ref- 1];  Mridha MK et al., 2021 [Ref- 2]; Singh MM, et al., 2017 [Ref-3]; Bhattarai D, et al., 2020 [Ref-4]]

**Author’s Response: **We agree with your comment and necessary changes have been made in the manuscript.

**Action taken in the manuscript: **Introduction has been revised with suggested reference.

  

- **Comment:** The methods of the study is very poor. The sample size estimation formula should write appropriately. The should report the scaling and scoring techniques of DAS 21.

**Author’s Response:  **We aim to conduct future studies with a robust methodology, incorporating the factors affecting DAS in our study Sample size estimation and DAS computation has been added in the manuscript.

**Action taken in the manuscript:  **Sample size formula has been explained in the methodology section of the manuscript in detail. DAS computation has been already added as per the suggestion in the previous comment.

  

- **Comment: **To enrich the Methods of the study, the authors are requested to follow any standard approach of writing Methods [e.g,. Haque M, et al., 2023 [Ref- 5]; Siddik M, et al., 2024 [Ref-1]].

**Author’s Response: **We agree with your comment. The suggested correction has been made.

**Action taken in the manuscript: **More details have been added to the methodology. The outcome measure has been described in detail as suggested by the reviewer, as demonstrated in the quoted sample studies.

  

- **Comment: **In the results section, the authors are strongly recommended to add inferential statistics to identify the associated risk factors of DAS among adolescents.

**Author’s Response: **The study of risk factors for DAS was outside the scope of our research, and therefore, no data on these factors was collected. We plan to incorporate this aspect into our future studies.

  

- **Comment: **The discussion should be revised according to the new results.

**Author’s Response: **As previously mentioned, no data was collected to study the factors associated with DAS. Therefore, it is not possible to add any new results. We will incorporate this aspect into future studies.

**Action taken in the manuscript: **Same has been added as the limitation of the study

# Reviewer report: Reviewer response for version 1

**DOI:** 10.5256/f1000research.152891.r257925

**Published:** 2024-03-23

**Authors:** Indranil Saha

**Affiliations:** 1 ICMR-Centre for Ageing and Mental Health (I-CAM), Indian Council of Medical Research, Kolkata, West Bengal, India

The manuscript needs following clarifications:

1. “p-values less than 0.05 were considered significant” – please correct as p value equal to or less than 0.05 was considered as statistically significant
2. As per rule the adolescents with completed 19 years should have been included as study participants
3. Colleges situated in urban and rural area: whether urban college truly represent urban adolescents? There may be so may students from rural area studying in urban college and the vice versa may be true.  
4. How socioeconomic status was measured in both the colleges?
5. Please edit the sample size formula – “n=4pq/D2” instead of 4, please mention as Z _∞_^2^.
6. In which language this DASS 21 scale was used? Was it validated and reliable in that settings?

Is the work clearly and accurately presented and does it cite the current literature?

Yes

If applicable, is the statistical analysis and its interpretation appropriate?

Yes

Are all the source data underlying the results available to ensure full reproducibility?

Yes

Is the study design appropriate and is the work technically sound?

Yes

Are the conclusions drawn adequately supported by the results?

Yes

Are sufficient details of methods and analysis provided to allow replication by others?

Partly

Reviewer Expertise:

Epidemiology, Non-communicable disease, Mental health

I confirm that I have read this submission and believe that I have an appropriate level of expertise to confirm that it is of an acceptable scientific standard, however I have significant reservations, as outlined above.

# Response

**Published:** 2024-04-09

**Authors:** Ajay Mallya

**Affiliations:** Community Medicine, Kasturba Medical College, Mangalore, karnataka, India

- Comment: “p-values less than 0.05 were considered significant” – please correct as p value equal to or less than 0.05 was considered as statistically significant

Author’s Response: We agree with your comment. The suggested correction has been made.

 Action Taken in the manuscript: The suggested correction has been made.

  

- Comment: As per rule the adolescents with completed 19 years should have been included as study participants

Author’s Response: We agree with your comment. We would like to clarify that adolescents who have completed 19 years were included in the study.

 Action Taken in the manuscript: we have mentioned that adolescents aged 19 years have included in the study in the methodology section.

  

- Comment: Colleges situated in urban and rural area: whether urban college truly represent urban adolescents? There may be so may students from rural area studying in urban college and the vice versa may be true.

Author’s Response:  In the data collection process, we ensured that the collected data is properly segregated into urban and rural categories based on the list of colleges obtained from the block education officer. However, we acknowledge that some participants may commute and study in a college that does not accurately represent the demography (urban and rural). This can be seen as a limitation of the study.

 Action Taken in the manuscript: Necessary changes have been done in the strengths and limitation section.

  

- Comment: How socioeconomic status was measured in both the colleges?

Author’s Response:  We have not measured the socioeconomic status of the participants in the study.

 Action Taken in the manuscript: As socioeconomic status was not included in the original study instrument we have not included it in the study.

  

- Comment: Please edit the sample size formula – “n=4pq/D2” instead of 4, please mention as Z∞ ^2^

Author’s Response: We agree with your observation.

 Action Taken in the manuscript: Necessary changes have been done in the manuscript.

  

- Comment: In which language this DASS 21 scale was used? Was it validated and reliable in that settings?

Author’s Response: DASS21 Scale was used in English in our study without any translation or alteration. As the students studying in PU college have English as their first language we used the scale without any translations. DASS21 scale in English is already validated among adolescents.

 Action Taken in the manuscript: We have duly incorporated this information into the methodology section of the manuscript for clarity.
